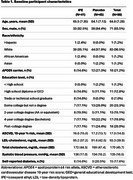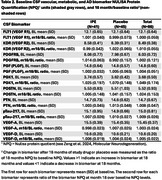# Impact of icosapent ethyl on CSF NULISA vascular, metabolic, and AD biomarkers in cognitively unimpaired Veterans at risk for dementia

**DOI:** 10.1002/alz70859_106943

**Published:** 2025-12-26

**Authors:** Cynthia M. Carlsson, Hannah Zylstra, Kate Cronin, Aleshia Cole, Elena Beckman, Allison C Eierman, Rachael E. Wilson, Richard J. Chappell, Carey E. Gleason, Henrik Zetterberg, Sterling C Johnson, Sanjay Asthana, Carol A. Van Hulle

**Affiliations:** ^1^ Wisconsin Alzheimer’s Institute, University of Wisconsin School of Medicine and Public Health, Madison, WI USA; ^2^ VA Geriatric Research, Education and Clinical Center (GRECC), William S. Middleton Memorial Veterans Hospital, Madison, WI USA; ^3^ Wisconsin Alzheimer's Disease Research Center, University of Wisconsin School of Medicine and Public Health, Madison, WI USA; ^4^ Division of Geriatrics and Gerontology, Department of Medicine, University of Wisconsin School of Medicine and Public Health, Madison, WI USA; ^5^ University of Wisconsin School of Medicine and Public Health, Madison, WI USA; ^6^ Wisconsin Alzheimer's Disease Research Center, University of Wisconsin‐Madison, School of Medicine and Public Health, Madison, WI USA; ^7^ Hong Kong Center for Neurodegenerative Diseases, Hong Kong, Science Park China; ^8^ UK Dementia Research Institute at UCL, London United Kingdom; ^9^ Department of Psychiatry and Neurochemistry, Institute of Neuroscience and Physiology, The Sahlgrenska Academy, University of Gothenburg, Mölndal Sweden; ^10^ Department of Medicine, University of Wisconsin‐Madison School of Medicine and Public Health, Madison, WI USA

## Abstract

**Background:**

Veterans Affairs (VA)‐eligible US military Veterans have increased susceptibility to Alzheimer’s disease (AD) dementia, possibly due to vascular contributions to AD pathology. The omega‐3 fatty acid eicosapentaenoic acid (EPA) has beneficial anti‐inflammatory and anti‐atherogenic properties, reduces clinical cardiovascular events, and has been associated with reduced risk for dementia. It is unclear if treatment with high‐dose EPA icosapent ethyl (IPE) improves cerebrospinal fluid (CSF) markers of vascular and metabolic health in adults at risk for AD.

**Method:**

The Brain Amyloid and Vascular Effects of Eicosapentaenoic Acid Study (BRAVE‐EPA, NCT02719327) recruited 131 cognitively unimpaired VA‐eligible Veterans from the Madison VA Hospital to participate in an 18‐month, placebo‐controlled, double‐blind randomized controlled trial of icosapent ethyl (IPE, Vascepa®, Amarin Corp.) 4g daily vs placebo. At baseline, month 9, and month 18, participants had cerebrospinal fluid (CSF) collected, MRI, and cognitive testing. In an exploratory analysis, CSF samples were analyzed at the University of Wisconsin ADRC Biomarker Lab using the multiplex NUcleic acid‐linked Immuno‐Sandwich Assay (NULISA)seq CNS disease panel kit according to manufacturer instructions. NULISAseq CNS disease panel includes measures of the vascular endothelial growth factor (VEGF) family, reflecting endothelial function, angiogenesis, vascular permeability, inflammation, and oxidation. ANOVA was used to test the ratio of 18 mo/baseline NULISA protein quantification (NPQ) for each biomarker.

**Result:**

Of the 131 recruited Veterans (ages 50‐76), 86 had CSF available at both baseline and month 18 for NULISA analyses. Baseline participant characteristics are noted in Table 1. This cognitively unimpaired sample was predominantly male, White, and demonstrated a breadth of vascular risk and educational attainment. Compared to placebo, 18 months of IPE therapy did not alter CSF biomarkers of vascular or metabolic health or a marker of AD pathology (pTau‐217)(Table 2, *p*‐value range 0.10 ‐ 0.98).

**Conclusion:**

In this cohort of cognitively unimpaired VA‐eligible Veterans, IPE did not alter NULISA CSF biomarkers of vascular and metabolic health or AD pathology (pTau‐217). While EPA has previously been shown to improve VEGF‐mediated inflammatory and atherogenic processes, further studies are needed to clarify the potential role of IPE or other omega‐3 fatty acids to alter AD risk through vascular risk modification.